# Epistasis between *COMT* and *MTHFR* in Maternal-Fetal Dyads Increases Risk for Preeclampsia

**DOI:** 10.1371/journal.pone.0016681

**Published:** 2011-01-31

**Authors:** Lori D. Hill, Timothy P. York, Juan P. Kusanovic, Ricardo Gomez, Lindon J. Eaves, Roberto Romero, Jerome F. Strauss

**Affiliations:** 1 Department of Obstetrics and Gynecology, Virginia Commonwealth University School of Medicine, Richmond, Virginia, United States of America; 2 Virginia Institute for Psychiatric and Behavioral Genetics, Virginia Commonwealth University School of Medicine, Richmond, Virginia, United States of America; 3 Department of Human and Molecular Genetics, Virginia Commonwealth University School of Medicine, Richmond, Virginia, United States of America; 4 Perinatology Research Branch, National Institute of Child Health and Human Development, National Institutes of Health, Department of Health and Human Services, Bethesda, Maryland, and Detroit, Michigan, United States of America; 5 Department of Obstetrics and Gynecology, Sótero del Río Hospital, Santiago, Chile; 6 Department of Obstetrics and Gynecology, Pontificia Universidad Católica de Chile, Santiago, Chile; Institute of Zoology, Chinese Academy of Sciences, China

## Abstract

Preeclampsia is a leading cause of perinatal morbidity and mortality. This disorder is thought to be multifactorial in origin, with multiple genes, environmental and social factors, contributing to disease. One proposed mechanism is placental hypoxia-driven imbalances in angiogenic and anti-angiogenic factors, causing endothelial cell dysfunction. C*atechol-O-methyltransferase* (*Comt*)-deficient pregnant mice have a preeclampsia phenotype that is reversed by exogenous 2-methoxyestradiol (2-ME), an estrogen metabolite generated by COMT. 2-ME inhibits Hypoxia Inducible Factor 1α, a transcription factor mediating hypoxic responses. COMT has been shown to interact with methylenetetrahydrofolate reductase (MTHFR), which modulates the availability of S-adenosylmethionine (SAM), a COMT cofactor. Variations in *MTHFR* have been associated with preeclampsia. By accounting for allelic variation in both genes, the role of *COMT* has been clarified. *COMT* allelic variation is linked to enzyme activity and four single nucleotide polymorphisms (SNPs) (rs6269, rs4633, rs4680, and rs4818) form haplotypes that characterize COMT activity. We tested for association between *COMT* haplotypes and the *MTHFR* 677 C→T polymorphism and preeclampsia risk in 1103 Chilean maternal-fetal dyads. The maternal ACCG *COMT* haplotype was associated with reduced risk for preeclampsia (*P* = 0.004), and that risk increased linearly from low to high activity haplotypes (*P* = 0.003). In fetal samples, we found that the fetal ATCA *COMT* haplotype and the fetal *MTHFR* minor “T” allele interact to increase preeclampsia risk (*p* = 0.022). We found a higher than expected number of patients with preeclampsia with both the fetal risk alleles alone (*P* = 0.052) and the fetal risk alleles in combination with a maternal balancing allele (*P*<0.001). This non-random distribution was not observed in controls (*P* = 0.341 and *P* = 0.219, respectively). Our findings demonstrate a role for both maternal and fetal *COMT* in preeclampsia and highlight the importance of including allelic variation in *MTHFR.*

## Introduction

Preeclampsia (PE) affects 5–8% of pregnancies worldwide and is characterized by hypertension and proteinuria after 20 weeks of gestation[1]. Although preeclampsia remains a significant source of maternal and perinatal mortality and morbidity, its etiology remains unclear. A genetic susceptibility to preeclampsia has been well established and genes involved with endothelial dysfunction, oxidative stress, angiogenesis and thrombophilia have been associated with preeclampsia[2]–[5].

It has long been recognized that preeclampsia is a placental disorder that results in the maternal syndrome. Placental hypoxia is a key feature of this condition and placentas from patients with preeclampsia show shallow trophoblast invasion[6]–[8] and failure of vascular transformation of the spiral arteries[9]–[11]. During normal placentation, oxygen levels tightly control the balance between angiogenic and anti-angiogenic factors to ensure adequate remodeling of the maternal spiral arteries and sufficient placental blood supply[12]. It is postulated that a hypoxia-driven disruption of the angiogenic balance causes the placenta to release factors that lead to intravascular inflammation[13]–[15], endothelial dysfunction[16]–[20] and the maternal phenotype. Indeed, abnormal concentrations of circulating angiogenic and anti-angiogenic factors including soluble fms-like tyrosine kinase (sFlt1), placental growth factor (PlGF), vascular endothelial growth factor (VEGF), transforming growth factor beta (TGF-β), and soluble endoglin (sENG) have been well documented in preeclampsia[12], [18], [19], [21]–[25]. Although abnormalities in these factors have been consistently demonstrated, there is no discernable pattern that characterizes preeclampsia, suggesting that a defect in an upstream regulator may contribute to the pathophysiology of preeclampsia.

2-methoxyestradiol (2-ME) is a natural metabolite of estradiol and it is generated by catechol-O-methyltransferase (COMT) in the placenta. 2-ME is a compound with diverse biological activities including inhibition of Hypoxia Inducible Factor 1α (HIF-1α)[26], [27]. HIF-1α is a transcription factor that mediates cellular responses to hypoxia and its expression is altered in preeclampsia[26]–[28]. Cytotrophoblastic invasion has also recently been reported to be modulated by 2-ME during hypoxic conditions[29]. In collaboration with Kanasaki *et al.*, we found that the *Comt*-deficient pregnant mouse exhibits a preeclampsia phenotype similar to that found in human preeclampsia, including hypertension, proteinuria and vascular and placental lesions; and the mouse preeclampsia-like phenotype is reversed by administration of 2-ME[27]. In this same report, circulating concentrations of 2-ME and placental COMT activity were significantly reduced in women diagnosed with preeclampsia, raising the possibility that altered production of 2-ME may contribute to the pathophysiology of preeclampsia by altering the placental response to hypoxia[27]. Moreover, severe preeclampsia and fetal growth restriction have been associated with reduced placental COMT activity[30], [31]. HIF-1α is an upstream regulator of many of the factors implicated in the angiogenic balance and endothelial dysfunction[12], [28]. By modulating HIF-1α activity, COMT represents a point at which this upstream regulator could be disrupted.

Human allelic variation in *COMT* has been associated with changes in enzyme activity levels[32], [33]. COMT is one of several enzymes that degrades catecholamines and is involved in vascular and metabolic homeostasis, including dopamine, epinephrine, norepinephrine, and catechol estrogens. The COMT enzyme is involved in a wide variety of physiological processes, such as prefrontal cortex function and lipid metabolism, and has been implicated in diseases such as schizophrenia, pain sensitivity, Parkinson's disease, and cancer[33]–[37]. Previous studies investigating the role of genetic variation in *COMT* have largely focused on the single nucleotide polymorphism (SNP) rs4680 Val^158^Met, which has been associated with a modest 4-fold difference in activity[32]. However, a recent functional analysis of four SNPs, rs6269, rs4633, rs4818, and rs4680, demonstrated that enzymatic activity is more precisely determined by three haplotypes of these SNPs, which result in a 25-fold difference in enzyme activity[33].

Preeclampsia is thought to be multifactorial in origin with multiple genes, environmental, and social factors acting in conjunction to cause disease[38]–[40]. Variations in the methylenetetrahydrofolate reductase (*MTHFR*) gene have been associated with elevated homocysteine, a risk factor for endothelial dysfunction, vascular disease, and preeclampsia[41]–[44]. Some previous studies have shown allelic variations in *MTHFR* to be associated with preeclampsia, although others have failed to replicate these associations[4], [45], [46]. MTHFR modulates the availability of methyl groups[44], which are the cosubstrate for COMT[32] and Roffman *et al.* recently showed that stratifying *COMT* genotypes by *MTHFR* genotype revealed a role of *COMT* in prefrontal cortex function[32], [44], [47].

In the present case-control study, we investigated the association between *COMT* haplotypes and preeclampsia in 1,103 Chilean maternal-fetal dyads. Haplotype frequencies were determined by genotyping 4 SNPs from the *COMT* gene: rs6269, rs4633, rs4818, and rs4680. Based on previous findings of haplotype-specific differences in enzymatic activity and protein levels, we evaluated the relationship of the functional variation linked to *COMT* haplotype and preeclampsia[33]. Finally, we assessed whether the relationship between *COMT* and preeclampsia was influenced by *MTHFR*.

## Results

Table 1 displays the demographic and clinical characteristics of mothers and neonates from pregnancies with preeclampsia as well as controls. No significant differences were observed in maternal age or neonatal sex between groups. Consistent with previous epidemiologic studies, patients with preeclampsia showed a significantly higher body mass index (BMI, *P*<0.001) and fewer previous live births (*P* = 0.007). In accordance with preeclampsia resulting in intrauterine growth restriction and indicated preterm birth, offspring born to women with preeclampsia showed a significantly lower gestational age at delivery and birthweight (*P*<0.001).

**Table 1:** Maternal and fetal characteristics of pregnancies diagnosed with preeclampsia and controls.

	Preeclampsia	Controls	*P*-value
Number of dyads	528	575	-
Maternal Age (*years*)	26.3 (7.5)	26.1 (6.2)	0.692
BMI (*kg/m^2^*)	26.4 (5.4)	24.5 (4.4)	<0.001
Previous live births	0.80 (1.19)	0.99 (1.08)	0.007
Birthweight (*grams*)	2805.7 (815.7)	3423.2 (303.0)	<0.001
Gestational age at delivery (*weeks*)	36.8 (3.4)	39.7 (1.1)	<0.001
Fetal sex (*% female*)	45.8	53.3	0.492

Data are presented as means (SD). BMI, body mass index.

Single SNP analysis revealed no associations between *COMT* polymorphisms rs6269, rs4633, rs4818, and rs4680 and preeclampsia in either maternal or fetal samples (Table S1). All SNPs were found to be in Hardy-Weinberg equilibrium in the maternal and fetal control samples separately. However, haplotype analysis showed the four SNPs to be in very high linkage disequilibrium (LD) for both maternal and fetal samples (Table S2). Three main haplotypes were identified: ACCG, ATCA, and GCGG (SNP order: rs6269, rs4633, rs4818, rs4680) and correspond to the low, intermediate, and high enzyme activities of COMT, respectively, identified by Nackley and colleagues[33].

The haplotype analysis of *COMT* frequency differences between cases and control subjects is shown in Table 2. A global test of differences among haplotypes reached statistical significance for maternal samples, but not for fetal samples (*P* = 0.016 and *P* = 0.116 respectively). Separate tests for haplotype-specific effects on disease class resulted in significant results for both the maternal (*P* = 0.004) and fetal (*P* = 0.038) ACCG haplotype (Table 2). This haplotype was observed more frequently in controls than cases for both maternal and fetal samples, indicating a possible protective effect. To control for the correlation of genotypes inherent in maternal-fetal dyads, we conditioned the maternal ACCG haplotype by the respective fetal ACCG haplotype. This resulted in only a significant effect of the maternal ACCG haplotype (maternal *P* = 0.041; fetal *P* = 0.446) on risk for disease and indicated that the effect of the ACCG haplotype was maternally derived and initial significant result for the fetal ACCG haplotype was likely a result of the correlation of the fetal-maternal genotype.

**Table 2:** *COMT* haplotype analysis for mothers and fetuses with and without preeclampsia.

Group	Haplotype	Frequency Preeclampsia	Frequency Controls	Chi-square	DF	*P*-value
Maternal	Global Test			8.260	2	0.016
	ATCA	0.373	0.348	1.531	1	0.216
	GCGG	0.310	0.277	2.807	1	0.094
	ACCG	0.317	0.375	8.112	1	0.004
Fetal	Global Test			4.308	2	0.116
	ATCA	0.381	0.359	1.302	1	0.254
	GCGG	0.302	0.283	0.907	1	0.341
	ACCG	0.318	0.360	4.308	1	0.038

*COMT* haplotype SNP order: rs6269, rs4633, rs4818, rs4680. DF, degrees of freedom. Maternal and fetal samples were analyzed separately. The Global test of association indicated that, in maternal samples, a significant difference in allele frequencies between cases and controls existed amongst the *COMT* haplotypes. When haplotypes were tested individually, both the maternal and fetal ACCG *COMT* haplotypes were found more frequently in controls than cases.

Additional multiple logistic regression analysis was performed to include risk factors for preeclampsia (maternal age, BMI, and previous live births). Results of a final regression model, which only included covariates, found to be significant in this population is shown in Table 3. Only the maternal ACCG haplotype (maternal *P* = 0.034, fetal *P* = 0.419) was observed to have a significant effect and was associated with a decreased risk of preeclampsia (OR = 0.796; 95% CI: 0.646, 0.982). Increased BMI was associated with an increased risk for preeclampsia (OR = 1.108; 95% CI: 1.076, 1.142) and a larger number of previous live births decreased the risk for preeclampsia (OR  = 0.782; 95% CI: 0.695, 0.880).

**Table 3:** Logistic regression model of primary risk factors for preeclampsia including presence of the ACCG *COMT* haplotype.

Term	Estimate (S.E.)	*P*-value	Odds Ratio (95% C.I.)
Maternal ACCG	−0.228 (0.107)	0.034	0.796 (0.646, 0.982)
Fetal ACCG	−0.092 (0.114)	0.419	0.912 (0.729, 1.140)
Maternal BMI	0.103 (0.015)	<0.001	1.108 (1.076, 1.142)
Previous live births	−0.246 (0.060)	<0.001	0.782 (0.695, 0.880)
Intercept	−2.289 (0.378)	<0.001	-

*COMT* haplotype SNP order: rs6269, rs4633, rs4818, rs4680. S.E., standard error; C.I., confidence interval; BMI, body mass index. When both maternal and fetal *ACCG* haplotypes from the maternal-fetal dyads were included in a single model, the maternal ACCG *COMT* remained significantly associated with reduced risk for preeclampsia. The fetal ACCG *COMT* haplotype is not associated with risk for preeclampsia after correcting for shared genetics between the mother and fetus.

Nackley et al. demonstrated in a mammalian expression system that *COMT* haplotypes resulted in an ordered progression of enzyme activity with the ACCG haplotype showing a 18–25 fold decrease in activity and the ATCA haplotype showing a 2.5–3 fold decrease in activity compared to the GCGG high activity haplotype[33]. Results of a multiple logistic regression model that included maternal and fetal terms to reflect enzymatic activity of the *COMT* haplotypes (*i.*e., each coded as an ordinal variable), maternal BMI, and previous live births are shown in Table 4. When maternal and fetal terms were analyzed separately, both show a significant positive relationship with increasing enzymatic activity and preeclampsia risk (*P* = 0.003 and *P* = 0.014 respectively). However, when both maternal and fetal terms were included in the same model, again the fetal association decreased in significance (*P* = 0.561) and the maternal ordered *COMT* haplotypes approached significance (*P* = 0.061).

**Table 4:** Logistic regression model of primary risk factors for preeclampsia including *COMT* haplotype specified according to reported enzymatic activity.

Term	Estimate (S.E.)	*P*-value	Odds Ratio (95% C.I.)
Maternal haplotypes *	0.166 (0.089)	0.061	1.180 (0.992, 1.406)
Fetal haplotypes †	0.052 (0.090)	0.561	1.053 (0.883, 1.257)
Maternal BMI	0.081 (0.019)	<0.001	1.084 (1.045, 1.126)
Previous live births	−0.236 (0.076)	0.002	0.790 (0.680, 0.917)
Intercept	−2.560 (0.529)	<0.001	

*COMT* haplotype SNP order: rs6269, rs4633, rs4818, rs4680. S.E., standard error; C.I., confidence interval; BMI, body mass index. Ordered *COMT* haplotypes: 1 = ACCG/ACCG, 2 = ACCG/ATCA, 3 =  ATCA/ATCA, 4 = ATCA/GCGG, 5 = GCGG/GCGG. Haplotypes were ordered from 1 (low activity) to 5 (high activity) in accordance with reported information on enzyme activity[33]. Maternal haplotypes showed increased risk for preeclampsia as haplotypes moved from low to high activity alleles.

*If maternal term fitted in model without fetal haplotypes *P* = 0.003, OR  = 1.221 (1.073, 1.390).

† If fetal term fitted in model without maternal haplotypes *P* = 0.014, OR  = 1.179 (1.034, 1.345).

Obesity is a major risk factor for preeclampsia and our results demonstrated BMI to be strongly associated with preeclampsia in this population[48]. COMT metabolizes catecholamines, which are known to modulate lipid mobilization[49]. Several studies have found modest associations between obesity and the rs4680 (Val^158^Met) SNP of *COMT* [50], [51]. The potential for COMT to contribute to preeclampsia risk through maternal BMI led us to investigate whether the association between maternal *COMT* haplotype and preeclampsia risk in our study could be explained by a relationship between *COMT* and BMI. PLINK was used to test for allelic associations between individual SNPs and BMI, where BMI was the quantitative phenotype. Haplotype frequencies were also generated in PLINK based on the four *COMT* SNPs and haplotype-specific tests were performed to test for frequency differences in association with BMI. Table 5 shows results for analyses that tested the relationship between maternal *COMT* and BMI in our study population. No significant associations between C*OMT* haplotypes or individual maternal SNPs and BMI were observed.

**Table 5:** Maternal *COMT* analysis for body mass index.

*COMT*		Estimate (S.E.)	*P*-value
SNP	rs6269	0.120 (0.231)	0.605
	rs4633	−0.182 (0.222)	0.413
	rs4818	0.092 (0.234)	0.693
	rs4680	−0.192 (0.223)	0.390
Haplotype	ATCA	−0.191*	0.392
	GCGG	0.114*	0.628
	ACCG	0.093*	0.675

Estimate is reported with (Standard Error) for SNPs.

* Standard errors are not calculated for haplotypes by PLINK. *COMT* haplotype SNP order: rs6269, rs4633, rs4818, rs4680. S.E., standard error; SNP, single nucleotide polymorphism.

The potential for *MTHFR* to influence risk for preeclampsia both through a single gene effect and an interaction with *COMT* was studied[4], [45], [47]. The rs4680 loci of *COMT* encodes for an amino substitution (Val^158^Met) and COMT protein with methionine at position 158 is reported to be less stable and with reduced activity[32]. However, this instability can be overcome by the binding of the methyl cosubstrate for COMT, s-adenosylmethionine (SAM)[52]. *MTHFR* modulates the availability of methyl substrates for COMT, including SAM, and the minor “T” allele of the rs1801133 SNP of *MTHFR* has been associated with reduced MTHFR activity and reduced production of SAM[44]. The ATCA haplotype of *COMT* is the only observed haplotype to have the “A” allele at the rs4680 loci and we postulated that an interaction between the ATCA haplotype of *COMT* and the minor “T” allele of the *MTHFR* rs1801133 SNP would result in a further decrease of COMT activity because there would not be adequate levels of SAM to stabilize the COMT protein. We therefore tested for epistasis between the ATCA *COMT* haplotype and SNP rs1801133 of *MTHFR*. Results of a multiple logistic regression model that included maternal and fetal terms for the interaction between the ATCA *COMT* haplotype and *MTHFR* are shown in Table 6. A significant interaction (*P* = 0.022) between the fetal ATCA *COMT* haplotype and the fetal *MTHFR* was observed, which resulted in an increased risk for preeclampsia (OR  = 1.370; 95% CI: 1.048, 1.792). The critical value for the test statistic associated with the interaction term was also estimated using permutation techniques and resulted in an empirical p-value of 0.023. No association was found between SNP rs1801133 in *MTHFR* and preeclampsia in either maternal or fetal samples (*P* = 0.470 and *P* = 0.225 respectively).

**Table 6:** Logistic regression model of *COMT-MTHFR* interaction risks for preeclampsia.

Term	Estimate (S.E.)	*P*-value	Odds Ratio (95% C.I.)
Maternal ACCG	−0.220 (0.126)	0.080	0.803 (0.627, 1.027)
Fetal ACCG	−0.126 (0.134)	0.345	0.882 (0.678, 1.146)
Maternal ATCA	0.017 (0.173)	0.921	1.017 (0.725, 1.428)
Fetal ATCA	−0.323 (0.174)	0.064	0.724 (0.515, 1.018)
Maternal MTHFR	−0.038 (0.143)	0.792	0.963 (0.727, 1.274)
Fetal MTHFR	−0.084 (0.145)	0.563	0.919 (0.692, 1.222)
Maternal ATCA: Maternal MTHFR	−0.028 (0.138)	0.840	0.972 (0.742, 1.274)
Fetal ATCA: Fetal MTHFR	0.315 (0.137)	0.022	1.370 (1.048, 1.792)
Maternal BMI	0.102 (0.015)	<0.001	1.107 (1.075, 1.140)
Previous live births	−0.252 (0.060)	<0.001	0.777 (0.691, 0.874)
Intercept	−2.082 (0.437)	<0.001	-

*COMT* haplotype SNP order: rs6269, rs4633, rs4818, rs4680. MTHFR SNP rs1801133. S.E., standard error; C.I., confidence interval; BMI, body mass index. An interaction between the fetal ATCA *COMT* haplotype and the minor “T” allele of *MTHFR* significantly increased the risk for preeclampsia; after correcting for risk factors identified to modulate risk in this population.

Our results revealed both a maternal protective effect and a fetal risk effect. Since our data included maternal-fetal dyads, we looked at the combination of maternal and fetal effects in a single pregnancy, focusing on the fetal high risk genotypes. Within cases we looked at the proportion of pregnancies that had two fetal high risk *COMT* ATCA x *MTHFR* “T” combinations with no maternal protective *COMT* ACCG allele and those that contained the two fetal high risk combinations with a balancing maternal *COMT* ACCG allele. We observed a higher than expected number of patients with preeclampsia with both the fetal risk alleles alone (Chi-square  = 3.789; *P* = 0.052) and the fetal risk alleles in combination with a maternal balancing protective allele (Chi-square  = 22.549; *P*<0.001). This non-random distribution across dyads was not observed in controls (*P* = 0.341 and *P* = 0.219, respectively).

## Discussion

Preeclampsia is a common disorder of pregnancy with potentially devastating complications[1], [53]. Placental hypoxia and endothelial cell dysfunction are central features of this disorder[12]. One proposed mechanism for preeclampsia is placental hypoxia-driven imbalance of angiogenic and anti-angiogenic factors[12], [18], [19], [21]–[24], resulting in endothelial dysfunction[16], [18]–[20], [54]. 2-Hydroxyestrogens are metabolized by COMT to produce 2-ME, a compound with diverse biological activities including inhibition of HIF-1α, a transcription factor that mediates cellular response to hypoxia[26]–[28]. Epidemiologic data has consistently demonstrated a strong genetic susceptibility to preeclampsia and *COMT* has been identified as a candidate gene for preeclampsia studies[2], [3], [27], [55]. In the present study, we found that the maternal ACCG haplotype of *COMT*, which is associated with low enzyme activity, was associated with a significantly reduced risk for preeclampsia in this population, and that the risk increased in a linear fashion from low to high activity alleles. We also found that epistasis between fetal *COMT* and *MTHFR*, which is associated with decreased enzyme activity as well, was associated with significantly increased risk for preeclampsia in this population.

We have previously reported that a *Comt* ^−/−^ mouse model exhibits a preeclampsia phenotype that is reversed by administration of 2-ME[27]. This model lead us to postulate that decreased production of 2-ME in humans, as a result of allelic variation in *COMT*, contributed to the development of preeclampsia[27]. The results of our current study showed that a maternal haplotype of *COMT*, which likely results in decreased levels of maternal 2-ME production, was in fact protective and decreased the risk for preeclampsia. In contrast, an interaction between a fetal haplotype of *COMT* and fetal *MTHFR*, which likely results in decreased level of fetal 2-ME production; increased the risk for preeclampsia as was initially predicted. A significant limitation to the *Comt* ^−/−^ mouse model is that COMT was absent in both the maternal and fetal compartments. By being deficient in both compartments, it is unclear whether the preeclampsia-like phenotype is a result of deficiencies in both compartments, or rather a deficiency in only one of the compartments. Although our results appear contradictory and do not support our initial hypothesis, we would like to propose that they are not inconsistent with the mouse model. We speculate that decreased maternal COMT activity would be beneficial by increasing the production of 2-ME by the placenta and that a placental loss of COMT activity is the key deficiency that contributes to the development of preeclampsia.

Previous research on genetic variation in the *COMT* gene has largely focused on a single SNP rs4680, which causes a valine to methionine substitution at position 158 (Val^158^Met) of the membrane bound version of the protein and position 108 of the soluble form. This amino acid substitution has been associated with a 4-fold decrease in activity in homozygote individuals[32]. Three additional SNPs, rs6269, rs4633, and rs4818, have recently been reported by Nackley *et al.* to contribute to haplotype structures with rs4680[33]. Although only rs4680 encodes an amino acid change, the additional polymorphisms are predicted to cause changes in mRNA secondary structure and thus, alter translation of the gene. Three main haplotypes were identified GCGG, ATCA, and ACCG and functional analysis in a mammalian expression system revealed changes in enzyme activity ranging from a decrease of 2.5–3 fold with the intermediate haplotype, ATCA, to a decrease of 18–25 fold with the low activity haplotype, ACCG[33]. Decreased activity of the low ACCG haplotype was attributed to low translation of the protein, while the decreased activity of the ATCA haplotype was attributed to impaired stability of the protein as a result an amino acid substitution at SNP rs4680[33]. Our study supports this conclusion in that we found the four SNPs to be in very high linkage disequilibrium and we identified the same three haplotypes in our Chilean population. Our single SNP analysis showed no significant results, but haplotype analysis revealed a significant association between *COMT* and preeclampsia.

These results are in agreement with several recent studies that identified *COMT* haplotype associations with attention deficit hyperactivity disorder, pain sensitivity, and Parkinson's disease[56]–[58]. Even more compelling, however, is our finding that preeclampsia risk changed in a linear fashion when we ordered haplotypes by reported enzymatic function. The ATCA haplotype was between the ACCG and GCGG haplotypes, with the ACCG haplotype being associated with the lowest risk for preeclampsia, and the GCGG haplotype with the highest risk. This progressive risk supports the assertion by Nackley et al. that ATCA represents the intermediate activity haplotype, while ACCG and GCGG are the extremes[33]. The results reported herein have significant implications not only for research in preeclampsia, but also for future studies investigating genetic variation in the *COMT* gene. Our results suggest that investigating only the *COMT* rs4680 Val^158^Met polymorphism provides incomplete information because it fails to recognize haplotype structures, which account for larger variations in enzyme activity. *COMT* haplotypes therefore can provide clarification of the role of *COMT* alleles in disease. The identification of haplotypes which modulate enzyme activity to a greater degree than a single polymorphism might explain the sometimes contradictory results of previous genetic association studies with other common disease[59], [60].

When considering the *COMT* gene independently, our results show that the maternal low activity haplotype of *COMT*, ACCG, was associated with a significantly lower risk for preeclampsia. The lower activity of the ACCG *COMT* haplotype has been reported to be the result of changes in mRNA secondary structure that lead to decreased translation of COMT protein[33]. Thus, the protective maternal effect of *COMT* on the risk for preeclampsia is likely the result of a translational mechanism. This significant association between *COMT* and preeclampsia highlights the importance of this gene in preeclampsia, but does not support the causative mechanism suggested by the *Comt* knock out mouse. The finding of a protective effect of a low COMT activity haplotype may suggest that reduced catecholamine metabolism or 2-ME production in the maternal compartment spares the placenta from hypoxia. Decreased metabolism of catecholestrogens in the maternal compartment would increase the amount circulating through the placenta and increase the potential production of 2-ME in this compartment.

Obesity is a major risk factor for preeclampsia and increased BMI was highly correlated with increased risk for preeclampsia in our study[48]. COMT metabolizes catecholamines including dopamine, epinephrine, norepinephrine, and chatecholestrogens. Catecholamines modulate lipid mobilization by means of adipose tissue lipolysis[49]. Specifically, estrogen and androgen concentrations are involved in body fat regulation and estradiol appears to stimulate preadipocyte proliferation and differentiation. Additionally, 2-ME has been shown to inhibit preadipocyte proliferation and differentiation *in vitro* [49]. In 2004, Tworoger *et al.* found that the rs4680 SNP in *COMT* modestly affected exercise-induced fat loss and in 2008, Annerbrink *et al.* found that the rs4680 SNP was associated with increased waist-to-hip ratio and abdominal sagittal diameter[50], [51]. In our study, there was no association between maternal *COMT* haplotypes or individual SNPs and BMI (Table 5). While we can conclude that BMI was not driving the relationship between *COMT* and the risk for preeclampsia in the present study, it is not valid to extend these results past the current sample since individuals with preeclampsia are oversampled in a case-control study.

Our *COMT* x *MTHFR* interaction findings support a similar finding by Roffman *et al.* that the low COMT activity allele was associated with disrupted prefrontal cortex function only in the presence of a low MTHFR activity allele [47]. In our study, the ATCA haplotype of *COMT* increased the risk for preeclampsia when the fetus also carried a low activity allele of the *MTHFR* gene, characterized by the minor “T” allele at SNP rs1801133. Unlike the translational mechanism proposed to govern the maternal *COMT* effect, the mechanism for the *COMT* x *MTHFR* interaction is most likely to be post translational. The ATCA haplotype of *COMT* is the only haplotype that alters the amino acid sequence and it results in a thermodynamically unstable COMT protein. However, this instability can be overcome by the binding of its cosubstrate, S-adenosylmethionine (SAM)[52]. MTHFR modulates the availability of SAM and the minor “T” allele at SNP rs1801133 results in lower production of SAM[44]. Therefore, when the fetus carries the “T” allele of *MTHFR* and the ATCA haplotype of *COMT*, the instability of COMT is not rectified and lower COMT activity is realized.

The identification of a fetal genetic risk factor for preeclampsia is an important step in understanding the cause(s) of preeclampsia. The placenta is fetal tissue and our results strengthen the argument that primary defects in the placenta play a central role in the development of preeclampsia. Additionally, our findings are consistent with the observations of reduced placental COMT activity and suggest that loss of activity in the fetal compartment of the *Comt* ^−/−^ mice appears to be responsible for the development of disease in this model [27].

Our findings have demonstrated both protective and risk alleles for *COMT* in association with the risk for preeclampsia. By investigating maternal-fetal dyads, we were able to explore the implications of both, seemingly contradictory, associations in a single pregnancy. We found a disproportionately high number of cases with two fetal ATCA *COMT* x *MTHFR* “T” risk combinations and with the two fetal risk combinations and one maternal ACCG protective *COMT* allele. What is most striking about our results however is the much larger chi-square value for the preeclamptic pregnancies that have two fetal risk combinations and one balancing maternal protective allele versus only having two fetal risk combinations and no balancing maternal allele (chi-square 22.549 vs. 3.789). The more significant nonrandom distribution of women with preeclampsia with a maternal protective ACCG *COMT* allele suggests that when the fetus is at high risk, it is preferred to have a maternal protective ACCG *COMT* allele to potentially offset the risk to some degree. Consequently, these pregnancies may be more viable than pregnancies where the fetus is at high risk, but has no maternal protection from disease. This hypothesis might help explain the findings in the *Comt* ^−/−^ mouse model and the observation that women with preeclampsia have lower levels of circulating 2-ME [27]. The balancing combination of maternal and fetal *COMT* alleles results in low COMT activity in both the maternal and fetal compartments and this mimics the low COMT profile of the knockout mouse. Although our results appear consistent with the mouse model, further studies are needed to understand how COMT behaves differently in the maternal and fetal compartments to modulate the risk for preeclampsia.

Ethnic differences in preeclampsia have been identified. Increased rates of preeclampsia have been found in African American, Hispanic, and Asian women compared to white women, with African Americans having the highest rates[61]. Additionally, maternal-paternal ethnic discordance has been associated with an increased incidence[61]. These findings indicate that differences in genetic causes of preeclampsia may exist between ethnic groups. Global variation in allele frequencies for both *COMT* and *MTHFR* have also been demonstrated[44], [62]–[67]. Moreover, allele frequencies for both genes are known to not only differ among major ethnic categories such as European, Asian, and African American, but substantial variation has also been demonstrated in subpopulations of each[44], [62]–[67]. Ethnic variation in each gene raises the possibility that different alleles of *COMT* and *MTHFR* could contribute to preeclampsia risk in different racial groups. The Chilean population in this study has a genetic background most similar to Western Europeans, and in particular, those of Spanish decent (2002 census). Future studies among different ethnic populations are needed to determine if our results can be extended to other ethnic groups.

## Materials and Methods

### Ethics Statement

This study was conducted according to the principles expressed in the Declaration of Helsinki. The study was approved by the Institutional Review Board of the Virginia Commonwealth University School of Medicine (IRB # HM12520). All patients provided written informed consent for the collection of samples and subsequent analysis.

### Study design and population

A case-control study was initiated by searching our clinical database and bank of biological samples and included Hispanic women and their neonates in the following groups: 1) Cases – women with preeclampsia and their neonates (*n* = 528 dyads); and 2) Controls – women who delivered at term with a normal pregnancy outcome and their neonates (*n* = 575 dyads). Participants received obstetrical care at the Sótero del Río Hospital in Santiago, Chile (an affiliated of the Pontificia Catholic University of Santiago, Chile). Exclusion criteria included: (1) known major fetal anomaly or demise; (2) multi-fetal pregnancy; (3) serious maternal medical illness (renal insufficiency, congestive heart disease, etc.); (4) refusal to provide written informed consent; and (5) a clinical emergency, which prevented counseling of the patient about participating in the study, such as fetal distress or maternal hemorrhage. All women provided written informed consent before collection of the samples. The use of clinical data and collection and utilization of maternal and neonatal blood for research purposes was approved by the Institutional Review Boards of the Sótero del Río Hospital and the *Eunice Kennedy Shriver* National Institute of Child Health and Human Development, NIH, DHHS. Ethnically, the Chilean population is estimated at nearly 95% white and mestizo (mixed white and Amerindian); 3% Amerindian; and 2% other. Mixtures between the conquering Spaniards, largely Andalusians and Basques, and the Mapuches (Araucanians) produced the principle Chilean racial type (2002 census). There is no reported evidence to support differences in disease prevalence amongst Chileans and there is no evidence to support the presence of group structure within this population. Therefore, population stratification was determined to not be a source of potential bias in this study population.

### Clinical definitions

Preeclampsia was defined based on the presence of gestational hypertension (systolic blood pressure ≥140 mmHg and/or diastolic blood pressure ≥90 mmHg) and proteinuria (≥300 mg in a 24-hour urine collection, two or more dipstick measurement of 1+, or one or more dipstick measurement ≥2+) according to ACOG (1) and the National High Blood Pressure Education Program[1], [68]. Patients were considered to have a normal pregnancy outcome if they did not have any medical, obstetrical, or surgical complication, and delivered a term neonate (≥37 weeks) of appropriate birth weight for gestational age[69] without complications.

### Sample collection

Maternal blood samples were obtained from the mother at the time of enrollment in the protocol, and from the umbilical cord immediately after delivery before the detachment of the placenta. Samples were collected with a vacutainer into tubes containing EDTA. The plasma tubes were balanced and centrifuged at 1300 *g* for 10 minutes at 4°C to separate cellular components from clear plasma, and the samples were stored at −70°C until assay.

### DNA extraction

DNA was extracted from maternal and cord blood with a Qiagen Autopure using standard procedures (Qiagen).

### Genotyping

Single-nucleotide polymorphism analysis was performed using real-time allelic discrimination TaqMan assays (Applied Biosystems) with modifications. All PCR reactions contained 25–75 ng of DNA, 6.25 ul TaqMan Universal Master Mix (Applied Biosystems) (2x), 0.3 ul TaqMan Genotyping Assay (Applied Biosystems) (20x), and water for a final volume of 12.5 ul. Real-time PCR was performed on an ABI 7500 Fast Real-Time PCR Machine (Applied Biosystems) under the following conditions: 50°C for 2 min, 95°C for 10 min, and 40 cycles of amplification (92°C for 15 sec and 60°C for 1 min). For each cycle, the software determined the fluorescent signal from the VIC- or FAM- labeled probe (Applied Biosystems). Allelic discrimination for *COMT* was performed using TaqMan Genotyping assays C___2538746_1 for SNP rs6269, C___2538747_20 for SNP rs4633, C___2538750_10 for SNP rs4818, C__25746809_50 for SNP rs4680 (Applied Biosystems). Allelic discrimination for *MTHFR* was performed using TaqMan Genotyping assay C__1202883_20 for SNP rs1801133.

### Statistical Analysis

Fisher's exact tests implemented in the PLINK software[70] were used to test individual SNPs for allelic associations with case-control status and to confirm Hardy-Weinberg equilibrium in the control group only. Inter-SNP linkage disequilibrium calculations for *COMT* were performed in Haploview (version 4.0)[71]. Haplotype frequencies were also generated in PLINK based on the four *COMT* SNPs and both global and haplotype-specific tests were performed to test for frequency differences between disease status for maternal and fetal samples separately. Haplotypes with an independent effect were further investigated by multiple logistic regression in R to condition by covariates known to influence rates of preeclamsia and to adjust for the correlation between maternal-fetal genotypes. These tests involved assigning haplotypes to subjects based on the most likely phase reconstructed haplotypes generated by the expectation-maximization algorithm implemented in PLINK. An additive term for the haplotype of interest was coded as 0, 1, or 2 based on copy number present. Based on the previously mentioned haplotype-specific functional information from Nackley et al.[33], we also coded *COMT* haplotypes to reflect enzymatic activity. *COMT* haplotypes were sequentially ordered 1 through 5 where 1 was ACCG/ACCG, 2 was ACCG/ATCA, 3 was ATCA/ATCA, 4 was ATCA/GCGG, and 5 was GCGG/GCGG. Interactive effects between the maternal ATCA *COMT* haplotype and maternal *MTHFR* and the fetal ATCA *COMT* haplotype and fetal *MTHFR* were tested using multiple logistic regression in R. The *MTHFR* was included as an additive term coded as 0, 1, or 2 based on copy number of the minor “T” allele. Permutation analysis in R with 10,000 iterations was used to compare models with and without significant interaction terms. Logistic regression in R was used to test for differences in clinical characteristics between disease classes for non-genetic variables.

## Supporting Information

Table S1 *COMT* single SNP analysis for maternal and fetal samples with and without preeclampsia.
(DOC)

Table S2 *COMT* pair-wise SNP linkage disequilibrium analysis for maternal and fetal samples.
(DOC)
