# Supplementary material for: Epistasis between COMT and MTHFR in Maternal-Fetal Dyads Increases Risk for Preeclampsia
Source: PLoS One. 2011 Jan 31;6(1):e16681. doi: 10.1371/journal.pone.0016681 (PMC3031618; doi:10.1371/journal.pone.0016681)
Supplement: Table S1 — COMT single SNP analysis for maternal and fetal samples with and without preeclampsia. (DOC) [file pone.0016681.s001.doc]

**Table S1. *COMT*** single SNP analysis for maternal and fetal samples with and without preeclampsia.

| Group | *COMT* SNP | Frequency Preeclampsia | Frequency Controls | Chi-square | *P*-value |
| --- | --- | --- | --- | --- | --- |
| Maternal | rs6269 | 0.316 | 0.284 | 2.676 | 0.104 |
|  | rs4633 | 0.372 | 0.347 | 1.551 | 0.214 |
|  | rs4818 | 0.307 | 0.274 | 2.897 | 0.091 |
|  | rs4680 | 0.367 | 0.348 | 0.858 | 0.373 |
| Fetal | rs6269 | 0.308 | 0.287 | 1.142 | 0.305 |
|  | rs4633 | 0.376 | 0.355 | 1.040 | 0.309 |
|  | rs4818 | 0.299 | 0.283 | 0.705 | 0.425 |
|  | rs4680 | 0.376 | 0.354 | 1.154 | 0.288 |

SNP, single nucleotide polymorphism.
