# Supplementary material for: Epistasis between COMT and MTHFR in Maternal-Fetal Dyads Increases Risk for Preeclampsia
Source: PLoS One. 2011 Jan 31;6(1):e16681. doi: 10.1371/journal.pone.0016681 (PMC3031618; doi:10.1371/journal.pone.0016681)
Supplement: Table S2 — COMT pair-wise SNP linkage disequilibrium analysis for maternal and fetal samples. (DOC) [file pone.0016681.s002.doc]

**Table S2. *COMT*** pair-wise SNP linkage disequilibrium analysis for maternal and fetal samples.

| Group | *COMT* SNP Combination | D’ (95% C.I.) | R^2^ |
| --- | --- | --- | --- |
| Maternal | rs6269:rs4633 | 1.000 (0.98, 1.00) | 0.239 |
|  | rs6269:rs4818 | 0.991 (0.97, 1.00) | 0.936 |
|  | rs6269-rs4680 | 0.974 (0.93, 1.00) | 0.225 |
|  | rs4633:rs4818 | 0.987 (0.95, 1.00) | 0.222 |
|  | rs4633:rs4860 | 0.984 (0.97, 1.00) | 0.959 |
|  | rs4818:rs4680 | 0.980(0.94, 1.00) | 0.217 |
| Fetal | rs6269:rs4633 | 1.000 (0.98, 1.00) | 0.242 |
|  | rs6269:rs4818 | 0.984 (0.96, 1.00) | 0.940 |
|  | rs6269-rs4680 | 0.994 (0.96, 1.00) | 0.239 |
|  | rs4633:rs4818 | 0.994 (0.96, 1.00) | 0.232 |
|  | rs4633:rs4860 | 0.992 (0.98, 1.00) | 0.984 |
|  | rs4818:rs4680 | 0.994 (0.96, 1.00) | 0.232 |

SNP, single nucleotide polymorphism; D’, D prime between the two loci; C.I., confidence interval; R^2^, correlation coefficient between the two loci.
